# Chemosensory Anhedonia in Patients With Schizophrenia and Individuals With Schizotypy: A Questionnaire Study

**DOI:** 10.3389/fpsyt.2020.00481

**Published:** 2020-06-04

**Authors:** Zi-lin Li, Gao-jie Huang, Ze-tian Li, Shu-bin Li, Yi-le Wang, Jiu-bo Zhao, Jin-feng Wen, Thomas Hummel, Lai-quan Zou

**Affiliations:** ^1^Chemical Senses and Mental Health Lab, Department of Psychology, School of Public Health, Southern Medical University (Guangdong Provincial Key Laboratory of Tropical Disease Research), Guangzhou, China; ^2^Department of Psychology, Guangdong 999 Brain Hospital, Guangzhou, China; ^3^Smell and Taste Clinic, Department of Otorhinolaryngology, Technische Universität Dresden, Dresden, Germany; ^4^Department of Psychiatry, Zhujiang Hospital, Southern Medical University, Guangzhou, China

**Keywords:** schizotypy, schizophrenia, smell, taste, anhedonia

## Abstract

Anhedonia, the loss or decline of the ability to enjoy pleasure, is an important clinical characteristic of schizophrenia. Schizotypal traits refer to the appearance of subclinical symptoms of schizophrenia across normal people. Still, few studies have investigated chemosensory anhedonia in schizophrenia patients and schizotypy individuals. Seventy-one schizophrenia patients (SCZ), 162 schizotypy individuals (SCT) as selected by the Schizotypal Personality Questionnaire (SPQ), and 182 healthy controls (HC) participated in our study. We used the Positive and Negative Syndrome Scale (PANSS) to measure the clinical symptoms of schizophrenia patients. All participants completed the Chemosensory Pleasure Scale (CPS), which was used to assess participants’ smell and taste hedonic capacities. We found that the three groups differed in chemosensory anhedonia. The SCZ group presented more severe chemosensory anhedonia than the SCT group, and the SCT group presented more severe chemosensory anhedonia than the HC group. We also found that chemosensory hedonic capacity was negatively correlated with negative schizotypal traits in the SCT group. Our results suggested that chemosensory anhedonia is an important characteristic of schizophrenia spectrum disorders.

## Introduction

Anhedonia, the loss or decline of the ability to enjoy pleasure, is an important clinical characteristic of schizophrenia (1, 2). It has been consistently reported that schizophrenia patients presented severe physical and social anhedonia (3–5). Similar to the clinical symptoms of schizophrenia but in a weakened form, schizotypy is a group of behavioral traits that indicates the nature of schizotype and shows a high risk for schizophrenia (2, 6). More severe anhedonia was also reported in schizotypy individuals according to their performance on the Temporal Experience of Pleasure Scale, which means that they experienced relatively less pleasure overall (7). Specifically, according to previous studies, schizotypy individuals presented more severe social and physical anhedonia than healthy controls (5, 7–9). However, few studies have specifically investigated the chemosensory hedonic capacity of schizophrenia patients and schizotypy individuals.

Chemosensory hedonic capacities which refer to the ability to experience pleasure simulated by smell or taste are of great importance to the study of schizophrenia and schizotypy individuals. There is only one synapse between olfactory receptors and the olfactory cortex and there is no thalamic intermediary on olfactory processing, which may provide a direct processing pathway between sensory environment and brain areas (10, 11). In addition, there is an overlap in brain regions regulating olfactory function and emotion processing including the orbitofrontal cortex (OFC), amygdala, and hippocampus (12). Therefore, compared with other sensory stimuli, olfaction may be more sensitive in the detection of anhedonia. It has been found that olfactory function was negatively correlated with anhedonia in schizophrenia and schizotypy individuals (7, 9). The impairments of olfactory hedonic processing were also reported in schizophrenia (13). Additionally, increased negative symptoms in schizophrenia was related to limited range of olfactory preference ratings (14, 15) and increased olfactory anhedonia (12, 16).

There are two components of hedonic experience including anticipatory and consummatory pleasure which reflects anticipation for future pleasurable events and reaction to in-the-moment pleasure, respectively (17). Based on these two domains, the Temporal Experience of Pleasure Scale (18, 19) and the Anticipatory and Consummatory Interpersonal Pleasure Scale (20) were widely used to assess individual’s hedonic capacity. However, none of these scales specifically measure the ability to experience current or noncurrent chemosensory pleasure. To capture these components of anhedonia, Zhao et al. (21) developed the Chemosensory Pleasure Scale (CPS), a chemosensory hedonic measurement to assess smell and taste hedonic capacities. According to previous findings, schizophrenia patients mainly reported diminished anticipatory but not consummatory pleasure (22–25), which has been named the “emotional paradox” of schizophrenia (22). However, previous laboratory studies only investigated the current positive response (i.e., consummatory dimension) toward olfactory stimuli (26), so it is still unclear if schizophrenia patients also present the same “emotional paradox” in the chemosensory hedonic capacities. Therefore, we chose to use the CPS for a further investigation in the chemosensory anticipatory pleasure.

The aims of our study were to use the CPS to: 1) measure the differences in chemosensory hedonic capacities among schizophrenia patients (SCZ), schizotypy individuals (SCT), and healthy controls (HC); and 2) also examine the correlations between the chemosensory hedonic capacity and schizotypal traits or clinical syndromes. We hypothesized that the SCT group would report lower levels of chemosensory anhedonia than the SCZ group but both groups would present higher levels than the HC. Moreover, previous studies found that there was a relationship between the increased olfactory anhedonia and increased negative symptoms in schizophrenia (12, 16). Therefore, we also hypothesized that the ability to experience chemosensory pleasure was negatively associated with negative schizotypal traits or syndromes.

## Methods

### Participants

This study recruited three groups of participants including schizophrenia patients (SCZ), schizotypy individuals (SCT), and healthy controls (HC). Seventy-one schizophrenia patients including 15 first-episode patients (21.13%) (52 males, 19 females; age range 16 to 31 years, mean ± SD = 22.82 ± 3.82 years) were recruited from the Guangdong 999 Brain Hospital in Guangzhou, Guangdong, China. All the participants were greater than or equal to 16 years old, and also had greater than or equal to 5 years of education. They were all free from bad cold in recent week, history of nose or throat diseases, brain injury, drug abuse, and other neuropsychiatric disorders according to the oral reports from themselves, their families, or medical records. All the patients were diagnosed by licensed psychiatrists in accordance with the Diagnostic and Statistical Manual of Mental Disorders, 5th Edition (DSM-5) diagnostic criteria for schizophrenia (27). Their clinical symptoms were measured by the Positive and Negative Syndrome Scale (PANSS) (28). Excluding 13 patients without medication records and 2 patients without any antipsychotic medical treatments, a total of 56 patients were taking antipsychotic medications. Dosages of medication were converted into chlorpromazine equivalents (CPZ) (29) (see **Table 1**).

**Table 1:** Demographics and clinical characteristics of the participants.

	Schizophrenia group		Schizotypy group		Healthy controls		*F/χ^2^*	*Post-hoc*
	(n=71)		(n=162)		(n=182)			
	Mean	*SD*	Mean	*SD*	Mean	*SD*		
Age (years)	22.82	3.82	18.27	0.76	18.27	0.85	94.77***	HC, SCT<SCZ
Sex (F:M)	19:52		102:60		108:74		28.43***	HC, SCT>SCZ
Education (years)	9.79	2.76	–	–	–	–	–	–
PANSS							–	–
Positive symptoms	18.77	6.09	–	–	–	–	–	–
Negative symptoms	23.41	7.21	–	–	–	–	–	–
General psychopathology	44.19	12.04	–	–	–	–	–	–
Chlorpromazine equivalents(mg/day)	397.71	228.07	–	–	–	–	–	–

F, female; M, male; PANSS, Positive and Negative Syndrome Scale; SD, standard deviation. ***p < 0.001.

One hundred and sixty-two individuals with schizotypy (60 males, 102 females; age range 17 to 21 years, mean ± SD = 18.27 ± 0.76 years) and 182 controls (74 males, 108 females; age range 16 to 21 years, mean ± SD = 18.27 ± 0.85 years) were recruited from the Southern Medical University in Guangzhou, Guangdong, China. They were selected from a sample of 1,589 college students according to their performance on the Schizotypal Personality Questionnaire (SPQ) (30, 31). Participants scoring at the top and bottom 10% were considered as the SCT group (scored higher than 41) and the HC group (scored lower than 10), respectively (8, 9, 31). Base on their self-reports, they were all free from bad cold in recent week, history of nose or throat diseases, brain injury, drug abuse, and neuropsychiatric disorders.

Our study was approved by the Ethics Committees of Southern Medical University and Guangdong 999 Brain Hospital. All the participants were provided informed consent before our study began.

### Measures

#### Chemosensory Hedonic Traits

The Chemosensory Pleasure Scale (CPS) is a self-rating scale to assess individual’s abilities to enjoy smell and taste stimulation (21). It consists of 12 items capturing 3 factors: food, imagination, and nature which refers to “consummatory,” “anticipatory,” and “purely olfactory” dimensions, reflecting the hedonic capacities of eating, anticipating food, and smelling natural scents, respectively. Participants were required to report their pleasant experience of smell and taste based on the 6-point Likert scale (from 1 “very false for me” to 6 “very true for me”). A lower score on the CPS indicated more severe chemosensory anhedonia. The Cronbach’s α coefficient was 0.93 and the test-retest reliabilities of the CPS was 0.73.

#### Schizotypal Traits

The Schizotypal Personality Questionnaire (SPQ) is a self-rating scale for schizotypal personality disorder according to the DSM-III-R criteria (31). It contains 74 items capturing all nine schizotypal traits. It has a high internal reliability (0.91) and test-retest reliability (0.82). It can be used for screening for schizotypal personality disorder across normal people, and also for studying the correlation of individual schizotypal traits. In our study, we used the Chinese version of SPQ (30). It is a three-factor model including the cognitive-perceptual (also referred to as positive schizotypy), the interpersonal (also referred to as negative schizotypy), and the disorganized factors.

### Data Analysis

Data analysis was conducted with SPSS version 22 (SPSS Inc, Chicago, IL, USA). Descriptive statistics were used to characterize the SCT, SCZ, and HC groups separately. The normal distribution was assessed using Shapiro-Wilk normality test. Because of the non-normal distribution of the CPS data, the non-parametric *Kruskal*-*Wallis* test followed by Dunn-Bonferroni test for *post hoc* comparisons were used to analyze the group differences on CPS total and subscale scores. In addition, the effect size estimates of the *Kruskal*-*Wallis* test were calculated using the eta squared (ŋ^2^) measure. The formula of the ŋ^2^ estimates using H statistic was as follows (32):

$${ŋ}_{H}^{2}=\frac{H-k+1}{n-k}$$

As for the formula, the H value was the statistic of the *Kruskal*-*Wallis* test. The k and n value were the number of groups and the total number of participants, respectively.

Furthermore, we also calculated the correlations between the CPS and SPQ, PANSS, dose of medications as well as duration of illness. Pearson’s correlation analysis was used if the data was normally distributed, otherwise Spearman’s correlation analysis was conducted. The multiple correlation analyses were also corrected with the Bonferroni correction.

## Results

There were significant differences in age [*F* (2,412) = 94.77, *p* < 0.001] and sex (*χ*^2^ = 28.43, *p* < 0.001) among the three groups. The *post-hoc* comparison showed that the average age of SCZ group was significantly older than that of SCT group and HC group, while the SCT group and HC group had higher sex ratio than the SCZ group (see **Table 1**).

As shown in **Table 2**, the three groups differed significantly in the CPS total and three subscales scores (total: *H* =86.824, *p* < 0.001, ŋ^2^ = 0.21; food: *H* =37.612, *p* < 0.001, ŋ^2^ = 0.09; imagination: *H*=120.530, *p* < 0.001, ŋ^2^ = 0.29; nature: *H* =32.385, *p* < 0.001, ŋ^2^ = 0.07). *Post-hoc* comparisons found that the SCZ group presented significantly lower scores on the CPS and all the subscales than the remaining two groups. Besides, compared with the HC group, the SCT group showed significantly lower scores in the food subscale, but these two groups did not differ significantly in the CPS total scores, imagination and nature subscales.

**Table 2:** Chemosensory hedonic traits.

	Schizophrenia patients	Schizotypy group	Healthy controls	*H*	Effect size	*Pos-hoc*
	(n=71)	(n=162)	(n=182)		(ŋ^2^)	
	Median	Median	Median			
	(Min-max)	(Min-max)	(Min-max)			
CPS total	45 (12–70)	59 (12–72)	60 (30–72)	86.824***	0.21	HC, SCT>SCZ
Food	20 (5–30)	24 (5–30)	25 (12–30)	37.612***	0.09	HC>SCT>SCZ
Imagination	13 (4–23)	20 (4–24)	21 (11–24)	120.530***	0.29	HC, SCT>SCZ
Nature	12 (3–18)	14 (3–18)	15 (6–18)	32.385***	0.07	HC, SCT>SCZ

min, minimum score; max, maximum score; ***p < 0.001.

**Figure 1:**
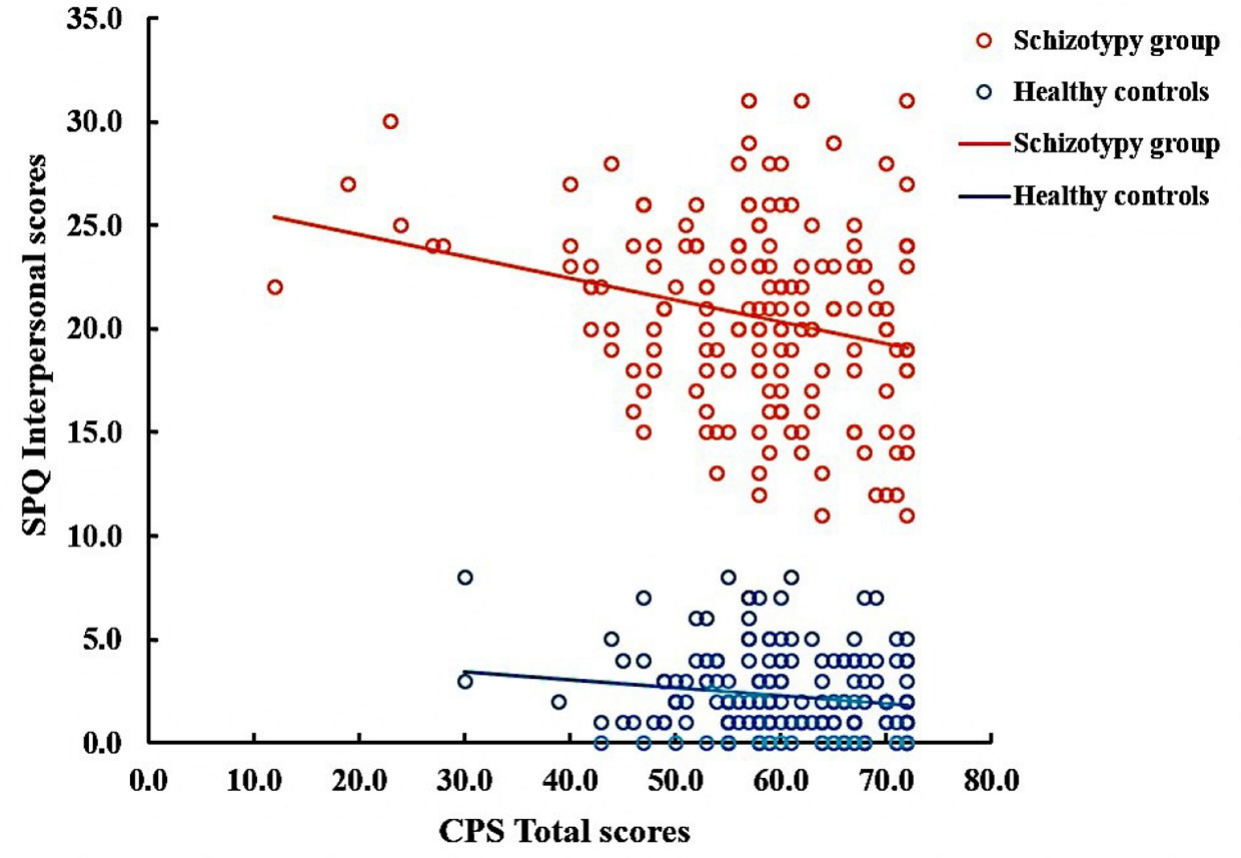
Correlation between Chemosensory Pleasure Scale (CPS) total scores and Schizotypal Personality Questionnaire (SPQ) Interpersonal scores in schizotypy group (*r* = −0.23, *p* < 0.01) and healthy controls (r=−0.14, *p*=0.063).

In the SCT group, the positive correlations were observed between the cognitive-perceptual dimension of SPQ and the CPS total score as well as the three subscales scores (total: *r* = 0.21, *p* < 0.01; food: *r* = 0.22, *p* < 0.01; imagination: *r* = 0.16, *p* < 0.05; nature: *r* = 0.19, *p* < 0.05). However, after Bonferroni correction, only the food subscale was significantly correlated with the cognitive-perceptual dimension (*p* < 0.006). The interpersonal dimension of SPQ was negatively correlated with the CPS total score (see **Figure 1**), the food and imagination subscales scores (total: *r* = −0.23, *p* < 0.01; food: *r* = −0.25, *p* < 0.01; imagination: *r* = −0.23, *p* < 0.01), but not with the nature subscale (*r* =−0.15, *p* > 0.05), and the correlations were still significant after Bonferroni correction (*p <* 0.006) (see **Table 3**). In the SCZ group, the negative syndromes subscale of PANSS had a significantly negative correlation with the food subscale of CPS (*r* = −0.25, *p* < 0.05), but this correlation was not found after Bonferroni correction (*p*>0.007). Besides, there were no significant correlations between the negative syndrome subscale of PANSS and the CPS total score, the imagination or nature subscales scores (*p*s > 0.05). The relationship between the CPS and the positive syndromes or general psychopathology of PANSS was not statistically significant (*p*s > 0.05) (see **Table 4**). Moreover, the CPS and all three subscales were not significantly correlated with the CPZ equivalence or duration of illness (*ps* > 0.05).

**Table 3:** Correlations between Chemosensory Pleasure Scale (CPS) and Schizotypal Personality Questionnaire (SPQ) scores in schizotypy.

	CPS	Food	Imagination	Nature	SPQ_T	SPQ_co_pe	SPQ_Int	SPQ_Diso
CPS	1							
Food	0.94**^,a^	1						
Imagination	0.88**^,a^	0.74**^,a^	1					
Nature	0.90**^,a^	0.77**^,a^	0.76**^,a^	1				
SPQ_T	−0.02	−0.02	−0.05	−0.01	1			
SPQ_co_pe	0.21**	0.22**^,a^	0.16*	0.19*	0.50**^,a^	1		
SPQ_Int	−0.23**^,a^	−0.25**^,a^	−0.23**^,a^	−0.15	0.60**^,a^	−0.13	1	
SPQ_Diso	<0.01	0.01	−0.02	−0.01	0.41**^,a^	−0.05	0.03	1

CPS, Chemosensory Pleasure Scale; SPQ_t, Schizotypal Personality Questionnaire total score; SPQ co_pe, SPQ cognitive-perceptual score; SPQ_Int, SPQ interpersonal score; SPQ_Diso, SPQ disorganized score; *p < 0.05; **p < 0.01, ^a^p < 0.006.

**Table 4:** Correlations between Chemosensory Pleasure Scale (CPS) and Positive and Negative Syndrome Scale (PANSS) in schizophrenia patients.

	CPS	Food	Imagination	Nature	PANSS_P	PANSS_N	PANSS_G
CPS	1						
Food	0.85**^,b^	1					
Imagination	0.83**^,b^	0.54**^,b^	1				
Nature	0.72**^,b^	0.46**^,b^	0.40**^,b^	1			
PANSS_P	0.05	−0.05	0.09	0.09	1		
PANSS_N	−0.18	−0.25*	−0.06	−0.14	0.16	1	
PANSS_G	0.06	−0.17	0.24	0.09	0.51**^,b^	0.51**^,b^	1

CPS, Chemosensory Pleasure Scale; PANSS, Positive and Negative Syndrome Scale; PANSS_P, PANSS positive syndromes score; PANSS_N, PANSS negative syndromes score; PANSS_G, PANSS general psychopathology score; *p < 0.05; **p < 0.01, ^b^p < 0.007.

## Discussion

This is the first study that directly used self-reported chemosensory pleasure scale to compare chemosensory hedonic capacity in schizophrenia patients, schizotypy individuals, and healthy controls. We found that the SCZ group presented more severe chemosensory anhedonia than the SCT group, and the SCT group presented more severe chemosensory anhedonia than the HC group. Additionally, chemosensory hedonic capacity was negatively correlated with negative schizotypal trait in SCT group.

Our finding of less pleasure from chemosensory stimulation in the SCZ group is consistent with previous neurobehavioral studies, which reported that SCZ patients having higher level of olfactory anhedonia than HC (12, 33). Moberg et al. (13) also found that hedonic odor processing was disrupted in schizophrenia patients. Moreover, we found that the schizophrenia patients differed from healthy controls in reports of current and noncurrent olfactory pleasure. They showed impairments in anticipatory and consummatory hedonic capacities. This finding was inconsistent with the previous findings that schizophrenia patients mainly reported intact consummatory but diminished anticipatory pleasure (22–25). Furthermore, schizophrenia patients reported the same levels of in-the-moment positive emotion as the healthy controls when processing laboratory stimuli (26, 34). The inconsistency of the results may further prove the uniqueness of olfaction. The overlapping brain regions between olfactory and emotion processing (12) and olfactory evolutionary significance in eating (35), selecting mates (36), and warning danger or threat (37) might make olfaction particularly sensitive to hedonic perception. For the individuals with schizotypy, previous studies consistently showed that they had significantly higher physical and social anhedonia (5, 7–9). Our study further investigated the chemosensory anhedonia in individuals with schizotypy. Just as the results of the studies on social and physical anhedonia, the SCT group showed more severe chemosensory anhedonia than the HC group. Our findings suggested that decreased chemosensory hedonic capacity may be an important trait marker in schizophrenia spectrum disorders.

In previous studies, it has been repeatedly found that negative symptoms were associated with olfactory deficits in schizophrenia patients (7, 38–41). In our study, although there were no significant differences between the SCT and HC on the CPS total, imagination, and nature subscales scores, we found that negative schizotypal trait was negatively correlated with the pleasant experience of smell and taste. This is consistent with previous studies which also reported that more severe negative symptoms were associated with a tendency to poorly evaluate hedonic experience of smell (12, 16, 42). It may suggest that chemosensory anhedonia was an important feature of negative schizotypal trait, but not positive schizotypal trait, which supports the idea that negative and positive schizotypy represent discrete dimensions (43). In the future, it may be important to further distinguish positive and negative schizotypy populations and compare their chemosensory hedonic capacities. Moreover, the food subscale was negatively correlated with the PANSS negative symptoms in the SCZ group, but this correlation was not found after Bonferroni correction. Besides, the significant negative correlations between negative symptoms and the CPS or the other two subscales scores were not observed in our study. The possible reason is that the clinical symptoms in our study were only measured by a single scale (i.e., PANSS), and the PANSS contains no items to measure anhedonia, unlike the Scale for the Assessment of Negative Symptoms (SANS) (44). The SANS and the Clinical Assessment Interview for Negative Symptoms (CAINS) (45) are sensitive and specific clinical measurements of negative symptoms in schizophrenia. In future studies, the SANS or CAINS should be used to measure the negative symptoms of schizophrenia in more details.

There were several limitations in this study. The age and sex of the SCZ group did not match well with the other two groups (the SCT and HC groups), so further research is needed to reduce the age and sex bias. Nevertheless, the SCT high-risk group showed higher levels of chemosensory anhedonia than the demographics well-matched HC group. In addition, participants did not rate the pleasantness in relation to the smelling of actual odors and or the eating of actual foods in our study, which should also be assessed in the future.

In summary, both patients with schizophrenia and schizotypy individuals showed more severe chemosensory anhedonia than health controls. Besides, the results also showed that chemosensory anhedonia was associated with negative schizotypal traits. Our findings further suggest that the decreased chemosensory hedonic capacity may be an important trait marker in schizophrenia spectrum disorders.

## Data Availability Statement

The datasets generated for this study are available on request to the corresponding authors.

## Ethics Statement

The studies involving human participants were reviewed and approved by Ethics Committees of Southern Medical University and Guangdong 999 Brain Hospital. Written informed consent to participate in this study was provided by the participants’ legal guardian/next of kin.

## Author Contributions

Z-LL and G-JH contributed equally to the study: they collected and analyzed the data, and wrote the first draft of the manuscript. Z-TL, S-BL and Y-LW collected and analyzed the data. J-FW collected and analyzed the data, and wrote the first draft of the manuscript. J-BZ and TH interpreted the data, and gave the comment to the first draft of the manuscript. L-QZ generated the idea, designed the study, interpreted the data, and wrote the first draft of the manuscript.

## Conflict of Interest

The authors declare that the research was conducted in the absence of any commercial or financial relationships that could be construed as a potential conflict of interest.

## References

[1] Horan WP Kring AM Blanchard JJ Anhedonia in schizophrenia: a review of assessment strategies. Schizophr Bull (2006) 32(2):259–73. 10.1093/schbul/sbj009 PMC2632206 16221997

[2] Meehl PE Schizotaxia, schizotypy, schizophrenia. Am Psychol (1962) 17(12):827. 10.1037/h0041029

[3] Blanchard JJ Mueser KT Bellack AS Anhedonia, positive and negative affect, and social functioning in schizophrenia. Schizophr Bull (1998) 24(3):413–24. 10.1093/oxfordjournals.schbul.a033336 9718633

[4] Burbridge JA Barch DM Anhedonia and the experience of emotion in individuals with schizophrenia. J Abnorm Psychol (2007) 116(1):30–42. 10.1037/0021-843X.116.1.30 17324014

[5] Wang Y Lui SS Zou LQ Zhang Q Zhao Q Yan C Individuals with psychometric schizotypy show similar social but not physical anhedonia to patients with schizophrenia. Psychiatry Res (2014) 216(2):161–7. 10.1016/j.psychres.2014.02.017 24589449

[6] Meehl PE Toward an integrated theory of schizotaxia, schizotypy, and schizophrenia. J Pers Disord (1990) 4(1):1–99. 10.1521/pedi.1990.4.1.1

[7] Zou LQ Zhou HY Lui SSY Wang Y Wang Y Gan J Olfactory identification deficit and its relationship with hedonic traits in patients with first-episode schizophrenia and individuals with schizotypy. Prog Neuropsychopharmacol Biol Psychiatry (2018) 83:137–41. 10.1016/j.pnpbp.2018.01.014 29371026

[8] Chan RC Wang Y Yan C Zhao Q McGrath J Hsi X A study of trait anhedonia in non-clinical Chinese samples: evidence from the Chapman Scales for Physical and Social Anhedonia. PloS One (2012) 7(4):e34275. 10.1371/journal.pone.0034275 22529910 PMC3328477

[9] Zou LQ Geng FL Liu WH Wei XH Jiang XQ Wang Y The neural basis of olfactory function and its relationship with anhedonia in individuals with schizotypy: An exploratory study. Psychiatry Res (2015) 234(2):202–7. 10.1016/j.pscychresns.2015.09.011 26404551

[10] Gottfried JA Smell: central nervous processing. Adv oto-rhino-laryngology (2006) 63:44–69. 10.1159/000093750 16733332

[11] Patel RM Pinto JM Olfaction: anatomy, physiology, and disease. Clin Anat (2014) 27(1):54–60. 10.1002/ca.22338 24272785

[12] Kamath V Moberg PJ Kohler CG Gur RE Turetsky BI Odor hedonic capacity and anhedonia in schizophrenia and unaffected first-degree relatives of schizophrenia patients. Schizophr Bull (2013) 39(1):59–67. 10.1093/schbul/sbr050 21616912 PMC3523921

[13] Moberg PJ Kamath V Marchetto DM Calkins ME Doty RL Hahn CG Meta-analysis of olfactory function in schizophrenia, first-degree family members, and youths at-risk for psychosis. Schizophr Bull (2014) 40(1):50–9. 10.1093/schbul/sbt049 PMC3885295 23641047

[14] Cumming AG Matthews NL Park S Olfactory identification and preference in bipolar disorder and schizophrenia. Eur Arch Psychiatry Clin Neurosci (2011) 261(4):251–9. 10.1007/s00406-010-0145-7 20820794

[15] Doop ML Park S On knowing and judging smells: identification and hedonic judgment of odors in schizophrenia. Schizophr Res (2006) 81(2-3):317–9. 10.1016/j.schres.2005.08.006 16181773

[16] Strauss GP Allen DN Ross SA Duke LA Schwartz J Olfactory hedonic judgment in patients with deficit syndrome schizophrenia. Schizophr Bull (2010) 36(4):860–8. 10.1093/schbul/sbn178 PMC2894582 19223658

[17] Kring AM Elis O Emotion deficits in people with schizophrenia. Annu Rev Clin Psychol (2013) 9:409–33. 10.1146/annurev-clinpsy-050212-185538 23245340

[18] Chan RC Shi YF Lai MK Wang YN Wang Y Kring AM The Temporal Experience of Pleasure Scale (TEPS): exploration and confirmation of factor structure in a healthy Chinese sample. PloS One (2012) 7(4):e35352. 10.1371/journal.pone.0035352 22530007 PMC3329425

[19] Gard DE Gard MG Kring AM John OP Anticipatory and consummatory components of the experience of pleasure: A scale development study. J Res Pers (2006) 40(6):1086–102. 10.1016/j.jrp.2005.11.001

[20] Gooding DC Pflum MJ The assessment of interpersonal pleasure: introduction of the Anticipatory and Consummatory Interpersonal Pleasure Scale (ACIPS) and preliminary findings. Psychiatry Res (2014) 215(1):237–43. 10.1016/j.psychres.2013.10.012 24210182

[21] Zhao JB Wang YL Ma QW Zhao JB Zhang XY Zou LQ The Chemosensory Pleasure Scale: A New Assessment for Measuring Hedonic Smell and Taste Capacities. Chem Senses (2019) 44(7):457–64. 10.1093/chemse/bjz040 31201424

[22] Strauss GP The emotion paradox of anhedonia in schizophrenia: or is it? Schizophr Bull (2013) 39(2):247–50. 10.1093/schbul/sbs192 PMC3576151 23328158

[23] Gard DE Kring AM Gard MG Horan WP Green MF Anhedonia in schizophrenia: distinctions between anticipatory and consummatory pleasure. Schizophr Res (2007) 93(1-3):253–60. 10.1016/j.schres.2007.03.008 PMC1986826 17490858

[24] Yan C Lui SSY Zou LQ Wang CY Zhou FC Cheung EFC Anticipatory pleasure for future rewards is attenuated in patients with schizophrenia but not in individuals with schizotypal traits. Schizophr Res (2019) 206:118–26. 10.1016/j.schres.2018.12.003 30545761

[25] Favrod J Ernst F Giuliani F Bonsack C [Validation of the Temporal Experience of Pleasure Scale (TEPS) in a French-speaking environment]. L’Encephale (2009) 35(3):241–8. 10.1016/j.encep.2008.02.013 19540410

[26] Cohen AS Minor KS Emotional experience in patients with schizophrenia revisited: meta-analysis of laboratory studies. Schizophr Bull (2010) 36(1):143–50. 10.1093/schbul/sbn061 PMC2800132 18562345

[27] American Psychiatric Association Diagnostic and statistical manual of mental disorders (DSM-5®) Washington, DC (2013). 10.1590/s2317-17822013000200017 24413388

[28] Kay SR Fiszbein A Opler LA The positive and negative syndrome scale (PANSS) for schizophrenia. Schizophr Bull (1987) 13(2):261–76. 10.1093/schbul/13.2.261 3616518

[29] Woods SW Chlorpromazine equivalent doses for the newer atypical antipsychotics. J Clin Psychiatry (2003) 64(6):663–7. 10.4088/jcp.v64n0607 12823080

[30] Chen WJ Hsiao CK Lin CC Schizotypy in community samples: the three-factor structure and correlation with sustained attention. J Abnorm Psychol (1997) 106(4):649–54. 10.1037//0021-843x.106.4.649 9358696

[31] Raine A The SPQ: a scale for the assessment of schizotypal personality based on DSM-III-R criteria. Schizophr Bull (1991) 17(4):555–64. 10.1093/schbul/17.4.555 1805349

[32] Tomczak M Tomczak E The need to report effect size estimates revisited. An overview of some recommended measures of effect size. Trends Sport Sci (2014) 21(1):19–25.

[33] Plailly J d’Amato T Saoud M Royet JP Left temporo-limbic and orbital dysfunction in schizophrenia during odor familiarity and hedonicity judgments. Neuroimage (2006) 29(1):302–13. 10.1016/j.neuroimage.2005.06.056 16099179

[34] Yan C Cao Y Zhang Y Song LL Cheung EF Chan RC Trait and state positive emotional experience in schizophrenia: a meta-analysis. PloS One (2012) 7(7):e40672. 10.1371/journal.pone.0040672 22815785 PMC3399884

[35] Luisier AC Petitpierre G Ferdenzi C Clerc Berod A Giboreau A Rouby C Odor Perception in Children with Autism Spectrum Disorder and its Relationship to Food Neophobia. Front Psychol (2015) 6:1830. 10.3389/fpsyg.2015.01830 26648891 PMC4664613

[36] Mahmut MK Croy I The role of body odors and olfactory ability in the initiation, maintenance and breakdown of romantic relationships - A review. Physiol Behav (2019) 207:179–84. 10.1016/j.physbeh.2019.05.003 31077678

[37] Santos DV Reiter ER DiNardo LJ Costanzo RM Hazardous events associated with impaired olfactory function. Arch otolaryngology–head Neck Surg (2004) 130(3):317–9. 10.1001/archotol.130.3.317 15023839

[38] Brewer WJ Pantelis C Anderson V Velakoulis D Singh B Copolov DL Stability of olfactory identification deficits in neuroleptic-naive patients with first-episode psychosis. Am J Psychiatry (2001) 158(1):107–15. 10.1176/appi.ajp.158.1.107 11136641

[39] Malaspina D Coleman E Goetz RR Harkavy-Friedman J Corcoran C Amador X Odor identification, eye tracking and deficit syndrome schizophrenia. Biol Psychiatry (2002) 51(10):809–15. 10.1016/s0006-3223(01)01319-1 PMC2981869 12007455

[40] Corcoran C Whitaker A Coleman E Fried J Feldman J Goudsmit N Olfactory deficits, cognition and negative symptoms in early onset psychosis. Schizophr Res (2005) 80(2-3):283–93. 10.1016/j.schres.2005.07.028 PMC3886553 16125904

[41] Ishizuka K Tajinda K Colantuoni C Morita M Winicki J Le C Negative symptoms of schizophrenia correlate with impairment on the University of Pennsylvania smell identification test. Neurosci Res (2010) 66(1):106–10. 10.1016/j.neures.2009.10.001 PMC4064297 19819272

[42] Auster TL Cohen AS Callaway DA Brown LA Objective and subjective olfaction across the schizophrenia spectrum. Psychiatry (2014) 77(1):57–66. 10.1521/psyc.2014.77.1.57 24575913

[43] Dinn WM Harris CL Aycicegi A Greene P Andover MS Positive and negative schizotypy in a student sample: neurocognitive and clinical correlates. Schizophr Res (2002) 56(1-2):171–85. 10.1016/S0920-9964(01)00230-4 12084431

[44] Andreasen NC The Scale for the Assessment of Negative Symptoms (SANS): Conceptual and Theoretical Foundations. Brit J Psychiat (1981) 155(S7):49–52. 10.1192/S0007125000291496 2695141

[45] Kring AM Gur RE Blanchard JJ Horan WP Reise SP The Clinical Assessment Interview for Negative Symptoms (CAINS): final development and validation. Am J Psychiatry (2013) 170(2):165–72. 10.1176/appi.ajp.2012.12010109 PMC3785242 23377637

